# Solvent Influence on Zeta Potential of Stationary Phase—Mobile Phase Interface

**DOI:** 10.3390/molecules27030968

**Published:** 2022-01-31

**Authors:** Mikołaj Dembek, Szymon Bocian, Bogusław Buszewski

**Affiliations:** Chair of Environmental Chemistry and Bioanalytics, Faculty of Chemistry, Nicolaus Copernicus University, 7 Gagarin St., 87-100 Toruń, Poland; m.dembek@doktorant.umk.pl (M.D.); bbusz@chem.umk.pl (B.B.)

**Keywords:** zeta potential, stationary phase, solvent, surface

## Abstract

Zeta potential is a surface characteristic formed on the solid surface and liquid interface. It is an interesting way to describe the surface properties of materials; thus, a series of four homemade polar embedded stationary phases that contain phosphate groups incorporated into hydrophobic ligands were investigated according to surface zeta potential. Measurements were carried out using Zetasizer Nano ZS for the stationary phases suspensions prepared in various solvent and solvent binary mixtures. The negative zeta potential values were obtained for most cases due to negatively charged residual silanols and phosphate groups. However, in some solvents: tetrahydrofuran, isopropanol, and toluene zeta potential are positive. Additionally, it was observed that the zeta potential seems to be independent of the type of silica gel used for the stationary phase synthesis.

## 1. Introduction

Silica gel is the most common support of stationary phases synthesis. Despite the modification procedure, some portions of residual silanols remain unreacted at all times. The bonding of hydrophobic or polar groups to the silica surface influences the surface properties, changing their polarity. It also influences the zeta potential of such a surface when it is in contact with a liquid or a liquid mixture [1,2,3].

The zeta potential is the potential of the electric field created by surface charges in the point from which the liquid phase can move either by pressure gradient or by the action of the outer electric field [1,4]. From the practical point of view, the zeta potential is the critical characteristic of the electric double layer. Thus, this parameter is essential in describing the mechanisms occurring at the surface where the stationary phase is in contact with the liquid mobile phase. The creation of an electric double layer takes place in each liquid chromatographic separation and influences the retention and selectivity of the separation.

From the stationary phase point of view, the zeta potential corresponds to the surface charge of the stationary phase. It is evident for ion chromatography where stationary phases possess charges [5]; however, it is observed even for octadecyl materials [6] as a result of ionized silanol that was not reacted due to the steric hindrance [7]. For polar embedded stationary phases, the presence of electronegative atoms, such as nitrogen and oxygen, the polarization, and donor-acceptor interactions, also influence the zeta potential values [8]. Incorporating polar functional groups into the nonpolar ligands induces their different polarization and changes the ionization of the silanols on silica particles. This action affects the results obtained from zeta potential measurements and varies depending on the type of surface modification performed [8,9,10].

The zeta potential is affected by the type of liquid in which the silica is immersed. Various solvents solvate the surface differently; they differ in polarization and other physicochemical properties. A significant amount of solvent (e.g., water) may adsorb on the surface of the silica gel support [11]. For example, adsorbed water molecules support the ionization of surface silanols. It leads to negative charging of the silica gel surface. A similar situation may be observed in other solvents that may solvates protons. In such a case, solvent properties may strongly influence the zeta potential of given materials. Additionally, it is postulated that zeta potential may be affected by the adsorption of ions from the solution, especially hydroxide ions [12].

According to DVLO theory, the main energy affecting the stability of a particle in solution is the potential energy consisting of the force of attraction and repulsion of particles between each other [13,14]. In a stable suspension, the attractive van der Waals forces and repulsive forces between the double adsorption layers of the particles are in equilibrium, and there is no coagulation of the particles. If the repulsive forces are insignificant, the particles will aggregate, and the suspension will not be stable. So, to ensure the stability of the suspension, it is necessary to take care of the dominant effect of the repulsion energy of the double adsorption layers between the particles. It is possible to provide by steric or electrostatic repulsion [15]. In the case of silica grains of 5 um size, where the particle surface is modified with the phosphodiester group and attached hydrophobic groups, it is difficult to speak about steric repulsion. Therefore, electrostatic repulsion will play an important role. It is connected with the silica surface’s physicochemical character and the solvent properties in which the suspension is prepared.

The performance of a chromatography column depends on many determinants. One of them is the quality of packing of the stationary phase bed in the column. This quality is affected by several different factors. Among them, one of the more important is the proper choice of slurry and packing solvent. In their work, Vissers et al. [16,17,18] confirm that the suspension’s stability is not necessarily related to the complete absence of aggregation. Various techniques to ensure stability can be found in the literature but mainly involve balancing the suspension density, chemical, and mechanical stabilization, or the use of high or low viscosity solvents [18]. Research confirms that the presence of particle aggregation in suspension while ensuring its stability is favorable [17,19]. Among other things, zeta potential measurements allow one to determine the stability of a suspension without determining whether or not aggregation occurs in solution [15,17].

During the last years, zeta potential measurements became one of the methods to characterize chemically bonded stationary phases [6,20]. The easiest way is to determine the zeta potential based on electrophoretic mobility measurement (µ). The calculation can be done using the Smoluchowski, Hückel, or Henry equation [4,21,22]. They differ by considering the ratio of the radius of the test particle to the thickness of the electric double layer (EDL)-κa. Henry’s equation is expressed as:

$$\mu =\frac{2}{3}\frac{\varepsilon_{r}\varepsilon_{0}\zeta }{\eta }\cdot F\left(\kappa a\right)$$

where μ is electrophoretic mobility, η is the viscosity of the solution, ε_r_ is the relative permittivity of the medium, ε_0_ is the absolute permittivity of vacuum, ζ is zeta potential, and F(κa) is Henry’s function. If the value of the Henry function F(κa) is 1.0, this means that κa is much smaller than 1, and therefore, the thickness of the electric double layer is much larger than the radius of the test particle. In this case, the Hückel equation is used.

$$\mu =\frac{2}{3}\frac{\varepsilon_{r}\varepsilon_{0}\zeta }{\eta }$$

On the opposite side of the calculation, we have a situation in which the particle’s radius is much larger than the thickness of the electric double layer so that κa is much larger than 1.0, and the Henry function F(κa) is equal to 1.5. The Smoluchowski mathematical approximation is then used.

$$\mu =\frac{\varepsilon_{r}\varepsilon_{0}\zeta }{\eta }$$

In an intermediate situation, i.e., a kappa value slightly greater or less than 1, calculations are used to precisely determine the Henry function. One of them could be the Ohshima approximation or the O’Brien calculation [23,24,25]. The size of the test particle in the approximation is known and constant. At the same time, the thickness of the electrical double layer depends on the type of solvent and mainly on the presence of ions in it, that is, the ionic strength. A higher concentration of ions decreases the thickness of EDL, while a low concentration makes this layer thicker. Of course, when ions are present in the solution, the pH of the suspension solution will also play a significant role [4,23,26].

In our study, particles of 5 µm size are used, suspended mostly in organic solvents. Therefore, it is difficult to talk about the presence of ions in solution and ionic strength. The main role of charge distribution near the surface and in bulk is performed by functional groups or atoms of molecules with free electron pairs. With such large particles as 5-micron silica grains and poorly ionized solvents, the use of the Smoluchowski equation for zeta potential calculations is justified [21,27]. The zeta potential value is calculated according to the formula:

$$\zeta =\frac{\mu \eta }{\varepsilon_{r}\varepsilon_{0}}$$

Various chromatographic packings were investigated according to their zeta potential in chromatographic conditions. Materials with chemically bonded alkyl groups, with and without end-capping, and other novel stationary phases were tested. Pure silica gels were compared with silica-hydride materials. [5,6,8,9,10,28,29]. In addition to chemically bonded ligands, which has the most crucial influence on zeta potential, other studies were performed: (i) the coverage density of bonded groups [6], (ii) the ionization of chemically bonded functionalities [5,6], (iii) the influence of electronegative atoms (polar groups) in the structure of bonded moieties [8], (iv) the impact of the mobile solution composition, pH of the solution, and ionic strength [2,9,10], (v) the formation of water enriched layer [30,31,32], and (vi) hydroxide ion adsorption on the stationary phase surface [33,34,35].

The stationary phase zeta potential was usually tested in methanol, acetonitrile, water, and its mixture in the previous works. However, it seems reasonable to check how different solvents influence the zeta potential of stationary phases. Thus, the goal of our study was to determine the zeta potential of four polar embedded stationary phases with different organic moieties in the presence of 16 different solvents and solvent mixtures. It allows obtaining information about slurry properties used for column packing. Although, as reported in the literature [36], theoretically, the zeta potential results are ideal at infinite sample dilution. In reality, there is a limit to the ratio of solvent molecules to measured particles, which depends on the particle size. There is also an upper limit of sample concentration which, if exceeded, makes the measurement of zeta potential more complicated; however, it is individual and depends on the type of sample, the size of grains, the light transmission through the sample, or the polydispersity of the particle [37]. In our study, the concentration was increased. It is known that the suspension’s aggregation and stability depend on the concentration of solute and particles [26,38]. So, increasing the stationary phase concentration in our study is due to practical aspect as high-concentration suspension is used for packing chromatography columns. Hence, the results obtained more adequately relate to the existing solutions used for column packing.

## 2. Results and Discussion

The stationary phases tested in the study contain a relatively low carbon load compared to typical reversed-phase materials. The first reason was that we wanted to obtain a relatively weakly hydrophobic stationary phase to allow the elution in purely aqueous conditions. The second reason is that ligand binding with an ionized (or at least highly polar) phosphate group does not allow high coverage due to electrostatic repulsion.

Sixteen different solvent and solvent mixtures were used in the study. Solvent and its mixtures, viscosities, dielectric constants, and refractive indexes are listed in Table 1. Data from the table were used for zeta potential determination using Zetasizer Nano ZS. The research aims, among other things, to determine the stability of stationary phase suspensions in solvents of different polarities and viscosity. These results will allow the appropriate selection of solvents for slurry preparation and the choice of packing solvent when packing chromatographic columns.

**Figure 1:**
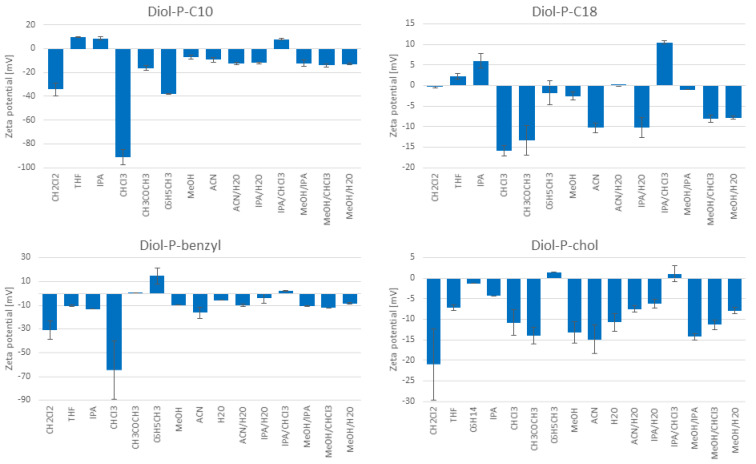
The zeta potential of stationary phases in different solvents with standard deviations (mV).

The stationary phases zeta potential measurement results in different solvents and solvent mixtures are presented in Figure 1. It has to be emphasized that there is no data on the plot for some solvents, mostly hexane (3) and water (10). It is a result of suspension instability that disallows the zeta potential measurement.

At first look, it can be seen that both positive and negative values of zeta potential were observed. In the case of octadecyl stationary phases (results published earlier [6]), independent of coverage densities, usually negative values were obtained regardless of the type of organic modifier (methanol or acetonitrile), or the water content in aqueous-organic solvent mixtures.

Negative zeta potential values indicate the accumulation of positive charges near the particle’s surface and, therefore, the electrically negative nature of the particle itself. In the case of phosphate embedded stationary phases tested in the study, negative zeta potential values may be caused by the partially ionized residual silanols and an ionized phosphate group. Residual silanol groups can possess different acidities [7,46]. More acidic with a pKa value between 3.5 and 4.6 are vicinal silanols. The single silanols are less acidic than vicinal silanols, with a pKa between 6.2 and 6.8 [46,47].

Since all tested stationary phases were synthesized on a silica support, a negatively charged surface will influence the zeta potential of all stationary phases. From the initial silanols on silica gel surface equaling 7.7 µmol/m^2^ determined in the previous study [48], around half are reacted or shielded by bonded ligands. Nevertheless, at least 3 µmol/m^2^ of silanols are available for interactions, and some of them can ionize while affecting the negative zeta potential values. Additionally, this effect may be enhanced by the presence of phosphates. However, different functional groups used for silica modification may weaken the influence of silanols on the silica support on the zeta potential due to different interactions with solvent molecules. In some solvents, tetrahydrofuran (2), hexane (3), isopropanol (4), and toluene (7), zeta potential values are positive. Positive values were also observed for all stationary phases in the isopropanol/chloroform mixture (13). It is observed mainly for the Diol-P-C10 and Diol-P-benzyl stationary phases. Also, the highest negative values were observed for chloroform for these materials.

From a suspension stability point of view, the criterium of zeta potential is higher than ±30 mV. If the value is lower than ±30 mV, the suspension may not be stable. In the data obtained, the values are usually lower than ±30 mV. Most of the results are in the range of ±20 mV. The most stable suspensions were obtained in chloroform, reaching up to −91 mV. As determined by zeta potential measurements, suspension stability does not imply a complete absence of aggregation. Suspended solids may aggregate into small aggregates while maintaining suspension stability. Therefore, the interpretation of the obtained results should lean toward the evaluation of suspension stability rather than statements determining the presence or absence of aggregates. Suspension stability can also be obtained by balancing the solvent and dispersed phase density or using a high viscosity solvent. Determining the stability of the suspension allows for a good choice of slurry solvent, which is one of the essential parameters when efficiently packaging chromatography columns.

Although it is known that zeta potential is not a direct measure of a surface charge, it may give some information, such as which surface attracts ions. However, we can explain the negative zeta potential values by negative charges on the surface. In theory, the highest negative charge should be observed in proton-acceptor solvents, e.g., water, which solvates protons and enhances the ionization of silanols and phosphates. The highest negative values are observed for chloroform, which does not meet these conditions. This case may be related to the non-zero dipole moment of the chloroform and its significant number of free electron pairs present at the chlorine atoms. Adsorption of chloroform molecules on the surface of the phase can cause high negative charge accumulation, which is manifested by high negative zeta potential values. It shows that values of zeta potential are difficult to predict without measurements.

Indirect correlation between surface charge and zeta potential explains the positive values obtained in measurement. Based on the stationary phases’ surface properties, there are no positive charges on them. Additionally, no acceptor atoms can accept protons such as nitrogen and provide a positive surface charge [8]. Thus, the positive values at the stationary phase with an embedded phosphodiester group and a nonionic solvent must be due to the accumulation of excess positively charged molecules on the particle surface. This occurs with reduced dissociation of free silanols and phosphate groups, which depends on the type of solvent. In this case, positive zeta potential values are caused by more complicated phenomena, such as solvation, accumulation of some ions from solution, etc. Positive zeta potential values were also obtained in our previous work [6]. The use of a hydrophobic phase (C18) and a protic solvent, such as methanol, nevertheless gave a positive zeta potential value. This confirms that some kind of phenomena occurring on the surface of stationary phase particles are more complicated and attempts to predict and describe them are only theoretical assumptions.

Another problem in comparison may be caused by stationary phase wettability that is somewhat different in various solvents. It has to be emphasized that the silica gel used in the study is porous, with a specific surface area of 320 m^2^/g. This surface is mainly located in pores, and the accessibility to these pores may be different, changing the solvents. Porosity causes energy inhomogeneity of the surface. According to Ståhlberg [49], the distance from the surface at which the electrostatic potential in the electric double-layer approaches zero may be as large as 30 nm. It means that the potential may be present in the total inner pore volume of particles commonly used in packed columns for HPLC with a pore diameter around 10 nm. When measuring electrophoretic mobility, the double electrical layer that forms at individual pore walls can overlap, causing “clogging” of the pore. Therefore, much of the charge can be compensated inside the pores of the particle or in the empty spaces between particles in aggregates. Only the part coming from ionized groups on the outer particle surface will be responsible for the electrophoretic mobility. Of course, it changes depending on the solution composition. Nevertheless, according to Smoluchowski’s theory, the zeta potential is a global value, referring to whole particles or aggregates.

**Figure 2:**
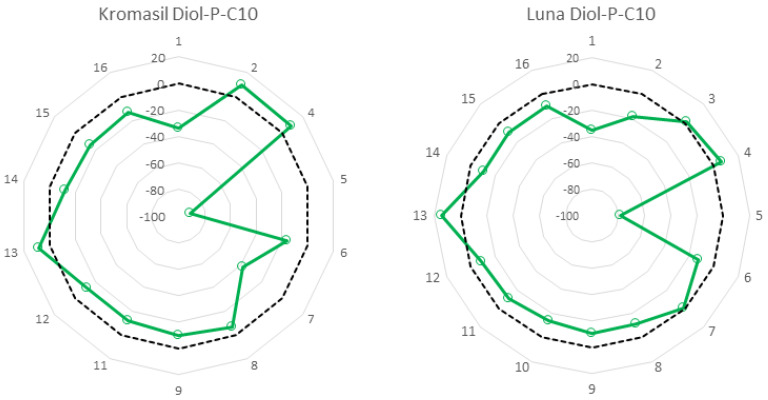
Comparison of zeta potential for stationary phases synthesized on a different support.

To interpret obtained results, the corresponding stationary phase was synthesized using a different support, Luna 100 Å instead of Kromasil 100 Å. The comparison of these two phases is presented in Figure 2. The shape of the radar plot for both stationary phases is very similar. The most crucial difference was that for Kromasil there was not possible to measure the zeta potential of the material in hexane and water suspensions (solvent no. 3 and 10 are omitted in the plot). It shows that zeta potential is reproducible if the surface physicochemical properties are similar. The convergence of the result trends is associated with the same surface modification. However, the different initial properties between Kromasil and Luna result in slight differences. The pure Kromasil and Luna silica zeta potential results on a DTS1070 capillary cell in pure water gave −45.8 ± 2.23 mV and −46.9 ± 0.78 mV, respectively. Numerically, these results cannot be compared with those obtained from measurements using a dip cell. On the other hand, comparing them indicates that the type of silica support used does not significantly affect the zeta potential value, so the surface modification influences the differences between the values obtained for the Diol-P-C10 phase on Kromasil and Luna.

It has to be emphasized that the similarities observed are in character and value. A significant difference was observed only for tetrahydrofuran (2) and toluene (7). In the case of tetrahydrofuran, a positive value was observed on Kromasil Diol-P-C-10, and on Luna Diol-P-C-10, it was negative. Kromasil Diol-P-C-10 exhibits a significant negative value for toluene, whereas on Luna Diol-P-C-10, the value was almost 0. The slight differences between the results may be due to differences in the coverage density of the two base materials. The different amounts and activity of free silanols may affect solvent solvation processes at the surface of silica grains.

## 3. Materials and Methods

### 3.1. Materials

A series of polar embedded stationary phases that contain phosphate and hydrophobic functional groups were tested. As a support for the synthesis, the Kromasil 100 silica gel (Akzo Nobel, Bohus, Sweden) was used. For the comparison, some stationary phases were also synthesized on Luna (Phenomenex, Torrance, CA, USA). Detailed characteristic of silica gels used for synthesis is presented in Table 2.

**Figure 3:**
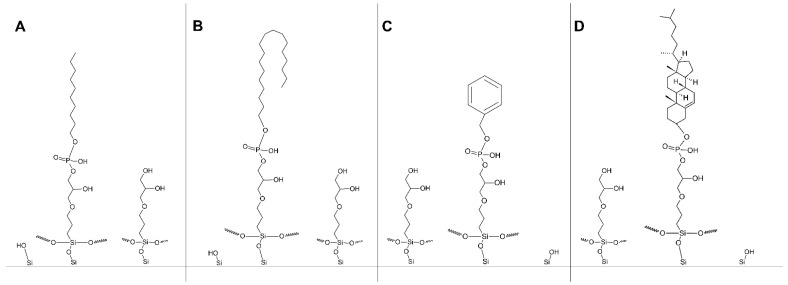
Schematic representation of stationary phase structures with embedded phosphodiester groups. (**A**) Diol-P-C10, (**B**) Diol-P-C18, (**C**) Diol-P-Benzyl, and (**D**) Diol-P-Chol. Each diagram also presents the structures of unreacted diol and residual silanols.

Four phosphodiester bonded stationary phases were tested. Structures of the materials are shown in Figure 3.

The properties of the stationary phases are listed in Table 3. Two Diol-P-C10 were synthesized on two different supports: Kromasil and Luna.

Reagents for the stationary phase synthesis: (3-glicidoxypropyl)trimethoxysilane, decanol, octadecanol, cholesterol, benzyl alcohol, and phosphoryl chloride were purchased from Alfa Aesar (Karlsruhe, Germany). Organic solvents used during synthesis: toluene, methanol, and hexane were ACS grade, purchased from Avantor Performance Materials (Center Valley, PA, USA).

### 3.2. Stationary Phase Synthesis

Before the chemical modification of silica gel, a sample of adsorbent was placed in a glass reactor protecting against the contact of the reagents with the external environment. Silica gel was dried at 180 °C under vacuum for 12 h. Then, the temperature was decreased to 90 °C, and (3-glicidoxypropyl)trimethoxysilane was added. After 12 h, the reaction products were washed out with toluene, methanol, and hexane and dried.

The obtained material was treated with 1% sulfuric acid to hydrolyze the epoxide ring in the second step. After the hydrolysis, the diol-bonded stationary phase was washed in water and methanol and dried under a vacuum.

Further, the diol-bonded silica was placed in a glass reactor and heated up to 100 °C for 10 h. Next, dried material was modified using phosphoryl chloride and proper alcohol: decanol, octadecanol, cholesterol, or benzyl alcohol to obtain Diol-P-C10, Diol-P-C18, Diol-P-Chol, and Diol-P-benzyl, respectively. Dried diol-modified silica was suspended in dry toluene. Next, the solution of phosphoryl chloride and alcohol was added. Reactions were carried out with the addition of triethylamine at 65 °C during 12 h under reflux. The reaction products were washed out with toluene, methanol, and hexane. Synthesized material was dried under a vacuum.

### 3.3. Instruments

The zeta potential measurement was performed using Zetasizer Nano ZS (Malvern Instruments, Malvern, UK) with a dip cell. Dip cell was equipped with a quartz cuvette. A Malvern DTS1070 capillary cell was used to measure suspensions of pure silica in water.

### 3.4. Methods

For each measurement, around 5 mg of the stationary bonded phase was suspended in 1 mL of solvent or solvent mixture using an ultrasonic bath for 10 min to help to obtain a stable suspension due to removing air from pores. Sample concentrations are increased over standard zeta potential measurements to make the results more relevant for the practical use of slurries in packing chromatography columns where the slurry concentration is high. The zeta potential measurement was done immediately after removing the suspension from the ultrasonic bath. The zeta potential measurement temperature was 25 °C. Each sample was measured three times, and the standard deviation was calculated from the results.

Before measurement, the stability of the suspension was routinely tested by Zetasizer. In the case of good suspension, the stationary bonded phase’s zeta potential in solutions was automatically calculated using Smoluchowski’s equation by Zetasizer from electrophoretic mobility. A detailed description was provided in the previous studies [5,6].

## 4. Conclusions

Five phosphate embedded stationary phases were tested according to the zeta potential in various solvents and solvent mixtures. The negative zeta potential values were obtained for most cases due to negatively charged residual silanols and phosphate groups. However, in some solvents, tetrahydrofuran, isopropanol, and toluene zeta potential get positive values, which may result from solvation phenomena and adsorption of ions from the solution. Additionally, it was observed that the zeta potential seems to be independent of the type of silica gel used for the stationary phase synthesis.

The results obtained will allow the appropriate selection of solvents to prepare stationary phase slurries used in the chromatography column packing procedure. As mentioned in the text, the appropriate choice of solvent is based on the formation of a stable slurry and the stationary phase. According to the work of Vissers [16,17,18], packing efficiency depends on both the aggregation of the stationary phase particles in the suspension and the stability of the slurry. For the most suitable choice to be made, research must continue in terms of aggregation evaluation using optical microscopy and chromatographic studies performed on the efficiency of columns packed with stationary phases from different slurry solvents. Such studies are planned to be performed by our team. The results will be able to more precisely answer the question regarding the proper solvent for packing columns with stationary phases with embedded phosphodiester groups.

## Figures and Tables

**Table 1:** Characteristic of solvents used in the study.

Solvent/ Mixture Number	Solvent/Solvent Mixture (1/1 *v*/*v*)	Viscosity µ [cP] (20 ˚C)	Dielectric Constant ε_r_ (20 °C)	Refractive Index RI	Literature
1	Dichloromethane	0.43	9.08	1.424	[39,40]
2	Tetrahydrofuran	0.55	7.60	1.407	[40]
3	Hexane	0.31	1.89	1.375	[40]
4	Isopropanol	2.86 (15 °C)	18.3 (25 °C)	1.377	[40]
5	Chloroform	0.85	4.81	1.446	[40]
6	Acetone	0.32	20.7 (25 °C)	1.359	[40]
7	Toluene	0.59	2.4 (25 °C)	1.496	[40]
8	Methanol	0.55	32.6 (25 °C)	1.329	[40]
9	Acetonitrile	0.37	37.5	1.344	[40]
10	Water	1.00	78.54	1.333	[40]
11	Acetonitrile/Water	0.81	58.02	1.336	[40,41]
12	Isopropanol/Water	2.58	48.42	1.355	[40,42]
13	Isopropanol/Chloroform	0.64	11.6	1.411	[40,43]
14	Methanol/Isopropanol	0.97	25.5	1.353	[40,44]
15	Methanol/Chloroform	0.65	18.7	1.383	[40,45]
16	Methanol/Water	1.54	78.5	1.340	[40,41]

**Table 2:** Characteristic of silica gels used in the study.

Parameter	Kromasil 100	Luna
Particle size [µm]	5	5
Specific surface area [m^2^/g]	320	400
Average pore size [nm]	11	10
Pore volume [cm^3^/g]	0.9	1.0

**Table 3:** Characteristic of stationary phases used in the study.

Stationary Phase	Carbon Load [%]	Coverage Density [µmol/m^2^]
Diol-P-C10	3.43	0.56
Diol-P-C18	4.18	0.42
Diol-P-Benzyl	2.86	0.56
Diol-P-Chol	9.31	0.87
Luna-P-C10	4.58	0.51

## Data Availability

The data presented in this study are available on request from the corresponding author.

## Sample Availability

Not available.
